# A comprehensive insight on the main physiological biochemical and related genes expression changes during the development of superficial scald in “Yali” pear

**DOI:** 10.3389/fpls.2022.987240

**Published:** 2022-09-02

**Authors:** Jingang He, Yunxiao Feng, Yudou Cheng, Meng Wang, Junfeng Guan

**Affiliations:** ^1^Institute of Biotechnology and Food Science, Hebei Academy of Agriculture and Forestry Sciences, Shijiazhuang, China; ^2^Key Laboratory of Hebei Plant Genetic Engineering Center, Shijiazhuang, China; ^3^Institute of Quality Standard and Testing Technology, Beijing Academy of Agriculture and Forestry Sciences, Beijing, China

**Keywords:** pear, superficial scald, phenolics, α-farnesene, ethylene

## Abstract

Superficial scald is a serious physiological disorder in “Yali” pear (*Pyrus bretschneideri* Rehd. cv. Yali) after long-term cold storage. Changes in superficial scald, ethylene production, α-farnesene and phenylpropane metabolism with associated gene expression in “Yali” pear treated with and without (control) 1-methylcyclopropene (1-MCP) were investigated. Compared with the control group (without 1-MCP), 1-MCP (1.0 μl L^–1^) significantly lowered the superficial scald index after 180 days of cold storage. During cold storage and shelf life, the contents of α-farnesene, conjugated trienols, chlorogenic acid, and epicatechin in the peel were reduced, while quercetin was enhanced in 1-MCP-treated fruit, and the expression of genes associated with ethylene synthesis (*ACS1*, *ACO1*), receptors (*ETR2*, *ERS1*) and signal transduction (*ERF1*), α-farnesene metabolism (*AFS1*, *HMGR2*, *GST7*), phenolic biosynthesis (*PAL1*, *C4H1*, *C4H2*, *HCT3*, *4CL2*, *C3H*), and oxidases (*PPO1*, *PPO5*, and *LAC7*) were significantly downregulated by 1-MCP. These results suggested that the onset and development of superficial scald was closely related to the ethylene receptor, conjugated trienols, chlorogenic acid and epicatechin and related genes expression in “Yali” pear.

## Introduction

After long-term cold storage, pears appear to develop superficial scald, which is similar to that in apples. Previous investigation have suggested that this phenomenon is related to the metabolism of α-farnesene and especially the accumulation of conjugated trienols (Whitaker et al., 2000, Whitaker, 2007, 2013; Lurie and Watkins, 2012; Zhou et al., 2017), and reactive oxygen species (ROS; Marc et al., 2020) as well as phenolic acids and flavonoids (Piretti et al., 1996; Zhi and Dong, 2018; Cebulj et al., 2020). 3-Hydroxy-3-methylglutaryl-CoA reductase (HMGR) and α-farnesene synthase (AFS) are key synthesis enzymes (Lurie and Watkins, 2012), and the *HMGR* and *AFS* genes expression is associated with α-farnesene and scald development (Gapper et al., 2006; Zhou et al., 2017). Furthermore, glutathione peroxidase (GPX) and glutathione S-transferase (GST) are suggested to promote the oxidation of α-farnesene to conjugated trienols (Whitaker, 2013), and the *GST* gene is involved in the development of superficial scald of pear (Zhou et al., 2017; Wang et al., 2018).

The phenolic acids and flavonoids are belonged to phenylpropanoid metabolites (Lurie and Watkins, 2012; Cebulj et al., 2020). Phenolic compounds are substrates of phenolic oxidation catalyzed by polyphenol oxidase (PPO), which is associated with the development of the scald (Busatto et al., 2014; Gao et al., 2015; Feng et al., 2018; Niu et al., 2018). Recent studies suggested that epicatechin oxidation catalyzed by laccase (LAC) is closely related to the development of scald in apples and pears (Gong et al., 2018; Zhai et al., 2021). However, the mechanism of the phenylpropanoid metabolism involved in the scald development has been less studied.

As an ethylene action inhibitor, 1-methylcyclopropene (1-MCP) could significantly inhibit the development of superficial scald by controlling the metabolism of ethylene, α-farnesene, phenolic acids, and flavonoids in apples and pears (Watkins, 2006; Wang, 2016; Busatto et al., 2018; Zhi and Dong, 2018; Larrigaudière et al., 2019), and 1-MCP suppress the expression of *AFS*, *HMGR*, *GPX*, and *GST* (Gapper et al., 2006; Xie et al., 2014, 2016; Zhou et al., 2017; Busatto et al., 2018; Karagiannis et al., 2018, 2020; Honaas et al., 2019; Marc et al., 2020) and the accumulation of ROS in fruit peel (Sabban-Amin et al., 2011). However, the correlation of the synthesis and signaling of ethylene with the α-farnesene accumulation, phenolic acids, and flavonoids should be fully explained, and the mechanism of 1-MCP controlling superficial scald in pear needs to be clarified in depth.

“Yali” pear is a famous cultivar in China, which has a large cultivation area and yield. However, it may appear superficial scald after long-term cold storage (more than 6 months), which severely affects the quality of the fruit’s appearance. The main aim of the present study was to investigate the development of scald in “Yali” pear treated with 1-MCP after cold storage under ethylene inhibition condition, and try to explore the correlation of ethylene synthesis, receptors and signal transduction with α-farnesene and main phenolic compounds metabolism in peels during the development of superficial scald in order to further reveal the roles of the main target substances associated with the α-farnesene, phenylpropane metabolism and the related genes in the onset and development of scald in “Yali” pear.

## Materials and methods

### Materials

“Yali” pear (*Pyrus bretschneideri* Rehd. cv. Yali) trees were selected in a commercial orchard located in Jinzhou, Hebei Province, China (N: 38°01′20.35′′, E: 115°04′23.94′′). Fruits were hand-harvested at September 27, 2017 and transported to the laboratory within 2 h and then uniformly sized fruit (fruit weight 286.76 ± 34.41 g) without damage or fungal infection were selected. After being stored overnight at room temperature (25 ± 1°C), one portion of the fruit was exposed to a final concentration of 1.0 μL L^–1^ 1-MCP (SmartFresh™, AgroFresh, Spring House, PA, United States) in an airtight container (60 L) with a circulation fan at 25°C for 24 h. Another portion of the fruit without 1-MCP was used as control group under the same condition. After treatment, pears were placed in paper boxes and stored in a fruit storage chamber (0 ± 0.5°C, RH = 90 ± 5%). After 60, 90, 120, 150, and 180 days of cold storage, pears were transferred to 20 ± 1°C for 1, 3, and 7 days shelf life experiment. Attributes such as fruit quality, superficial scald, ethylene production, and respiration rates were evaluated in triplicate, and ten fruit per replicate. Peel tissue was sampled and flash-frozen in liquid nitrogen (N_2_) and then stored at −80°C.

### Superficial scald index

Superficial scald index was classified according to the method reported by Calvo et al. (2015), the proportion of peel browning to total peel area was divided into four grades: grade 0 indicated no browning; grade 1 indicated 0% < browning area ≤ 25%; grade 2 indicated 25% < browning area ≤ 50%; and grade 3 indicated a browning area >50%.

The calculation formula is as follows: superficial scald index = Σ (browning grade × number of fruit per grade)/(total number of fruit × the highest grade).

### Fruit firmness and soluble solids content

Fruit firmness was determined using a digital fruit penetrometer (Model: GY-4, Top Instruments Co., Ltd., Hangzhou, China) at two equidistant points on the equatorial region with the skin removed. The firmness was expressed as N. Soluble solids content (SSC) was measured by a PAL-1 pocket digital refractometer (Atago Co., Ltd., Tokyo, Japan) for flesh juice squeezing from two equidistant points.

### Respiration and ethylene production rates

Respiration and ethylene production rates were measured in triplicate, and ten fruits were sealed in a 6 L container at 20°C as one replicate. After the fruit sealed for 1 h, 10 ml of gas was withdrawn from the container and injected into an HFY-1a CO_2_ infrared analyzer (Kexi Instrument Co., Ltd., Jiangsu, China) to measure the content of CO_2_. Subsequently, the respiration rate was calculated and expressed as the release rate for CO_2_ (ng kg^–1^ s^–1^). To measure ethylene production rate, 1 ml of gas was withdrawn from the container after the fruit sealed for 3 h, and then injected into the gas chromatograph (Model: GC9790II, Fuli Instruments Technology Co., Ltd., Wenling, China) equipped with a GDX-502 column and a flame ionization detector (FID). The temperatures of the column, vaporization oven and FID were set as 78, 120, and 200°C, respectively. N_2_ was used as the carrier gas with a rate of 40 ml min^–1^. The ethylene production rate was expressed as ng kg^–1^ s^–1^.

### Contents of α-farnesene and conjugated trienols

The contents of α-farnesene and conjugated trienols (CTols) were determined based on the method reported by Feng et al. (2018). A peel disk (1 cm in diameter) was formed along the equatorial part of the fruit, and 20 disks from each group were placed in a 25 ml graduated test tube. Afterward, 15 ml *n*-hexane was added and stored in the dark for 2 h. The extract was filtered by a clean Florisil SPE column, and the absorbance was recorded at 232, 281, and 290 nm. The concentration of α-farnesene and CTols were calculated using the molar extinction coefficients ε_232_ = 27740 for α-farnesene and ε_281–290_ = 25000 for the CTols, and the contents of α-farnesene and CTols expressed as nmol cm^–2^.

### Phenolic acid extraction

Extraction of phenolic acids was carried out according to the procedure reported by Wang et al. (2017). After liquid nitrogen grinding, 1 g of freeze-dried peel powder was added with 10 ml of 80% methanol containing 0.5% hydrochloric acid solution. The mixture was then ultrasonicated for 30 min and centrifuged at 10,000 × *g* for 10 min at 4°C, and the supernatant was collected finally. The extraction was repeated for three times. All the supernatants were combined and evaporated at 50°C under a gentle flow of nitrogen until completely dry. The residue was redissolved in 5 ml of 50% (v/v) methanol/ultrapure water and filtered through a 0.22 μm PTFE filter (Pall, MI, United States).

### Flavonoid extraction

Extraction of flavonoids was performed according to the method reported by Gao et al. (2019) with slight modifications. Two grams of peel powder mixing with 30 ml of 80% methanol was kept in the dark for 24 h at −20°C. Afterward, the mixture was centrifuged at 10,000 × *g* at 4°C for 10 min. Afterward the supernatant was filtered through a 0.22 μm PTFE filter (Pall, MI, United States) and collected for analysis immediately.

### UPLC–MS/MS analysis

The contents of the phenolic acids and flavonoids were performed according to the method described by Gao et al. (2019). The extracts were identified via an Acquity UPLC system (Waters, Milford, MA, United States) with a triple quadrupole mass spectrometer (TQ-S, Waters Micromass, Manchester, United Kingdom). The column used was an Acquity HSS C18 column (1.8 μm particle size; 2.1 mm × 150 mm; Waters, Milford, MA, United States). The column and sample managers were maintained at 40 and 10°C, respectively. Mobile phase A consisted of 0.1% (v/v) formic acid in water and mobile phase B consisted of 0.1% (v/v) formic acid in acetonitrile. The gradient used was as follows: 0.5–4.5 min, 5–30% B; 4.5–9.0 min, 30–90%; 9.0–10.0 min, 0.5% B. The mass spectrometer was operated in both positive and negative ionization modes depending on the structure and properties of compounds. The parameters were set as follows: capillary voltage, +2.5 kV/−1.0 kV; source temperature, 150°C; desolvation temperature, 500°C; cone gas flow, 150 L h^–1^; and desolvation gas flow, 1000 L h^–1^. Detection was conducted in multiple reaction monitoring (MRM) mode. The MS data were collected and analyzed by MassLynx™ 4.1 software (Waters, Milford, MA, United States). Quantification of the phenolic acids and flavonoids were performed using the standard curves with authentic standards in serial dilutions (1–500 ng mL^–1^).

### RNA isolation and real-time quantitative polymerase chain reaction analysis

After liquid nitrogen grinding, 100 mg peel powder was used for total RNA isolation by the RNA-prep Pure Plant Plus Kit (polysaccharides and polyphenolics-rich) (Tiangen Biotech Co., Ltd., Beijing, China). A total of 500 ng of RNA after elimination genomic DNA was used to generate the first strand cDNA via reverse transcription using a PrimeScript RT Reagent Kit with gDNA Eraser (Perfect Real Time) (TaKaRa Bio Inc., Dalian, China).

Real-time quantitative polymerase chain reaction analysis was performed on a Real-time System (ABI7500, Applied Biosystems, United States) using a TB Green™ Premix ex Taq II quantitative PCR kit (TaKaRa Bio Inc., Dalian, China). The sequences and references of primers for qRT–PCR are shown in Supplementary Table 1 (Fischer et al., 2007; Jugdé et al., 2008; Qian et al., 2014; Cheng et al., 2015; He et al., 2017; Gong et al., 2018; Li et al., 2018; Zhou et al., 2020; Zhai et al., 2021). The relative gene expression amount was calculated with the formula 2–ΔΔ*^Ct^* in Microsoft Excel 2007 (Livak and Schmittgen, 2001) by using *PbACTIN2* as the internal reference gene (Cheng et al., 2015).

### Statistical analysis

Duncan’s multiple comparison test was used to compare the significant differences between control and 1-MCP treatment (*P* < 0.05). Statistical analysis was performed via SPSS Statistics 23 (IBM Co., Armonk, NY, United States). Figures were generated by GraphPad Prism 8.0 (GraphPad Software, San Diego, CA, United States), heatmaps with clusters and PCA loading plots were created by Origin 9.0 (OriginLab Co., Northampton, MA, United States).

## Results

### Fruit quality and superficial scald index

**FIGURE 1:**
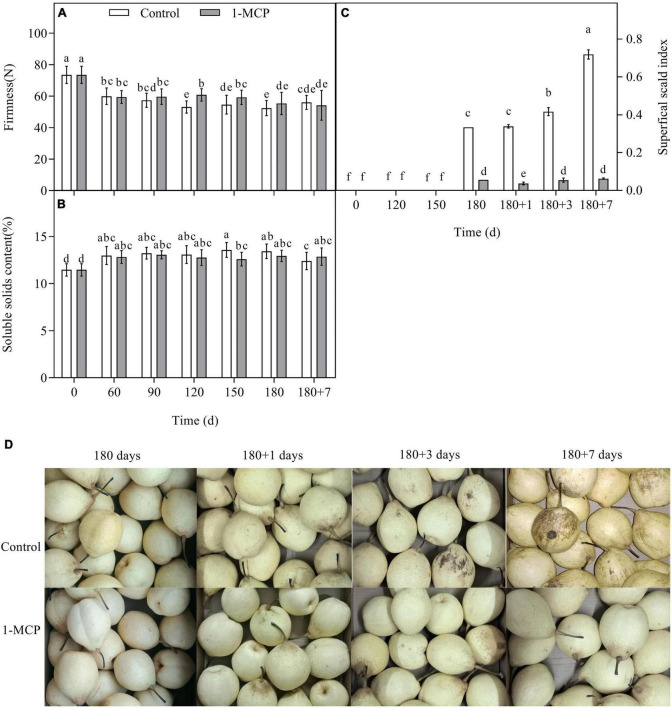
Effect of 1-MCP on firmness **(A)**, SSC **(B)**, superficial scald index **(C)**, and symptom **(D)** in “Yali” pear during cold storage and shelf life. 180 + 1, 180 + 3, 180 + 7 indicated Days 1, 3, 7 at shelf life after 180 days of cold storage, respectively. All data are expressed as means ± standard errors of triplicate samples. Different letters indicate significant differences (*P* < 0.05) by Duncan’s multiple comparison test.

Firmness and SSC were the main quality indicators, in which the firmness indicates the degree of fruit softening. During long-term cold storage, the firmness of the control fruit decreased, and the SSC increased. There was no obvious difference in the firmness and SSC between control and 1-MCP treatment fruit, except a higher firmness at Day 120 and a lower SSC at Day 150 in the 1-MCP treatment pears (Figures 1A,B). The superficial scald appeared after 180 days of cold storage, however, the scald index increased significantly in the control group during shelf life (Figures 1C,D). Nevertheless, the scald index was significantly reduced by 1-MCP and maintained at a much lower level (Figure 1C). 1-MCP had no significant effect on fruit firmness and SSC during cold storage and shelf life.

### Respiration and ethylene production rates

**FIGURE 2:**
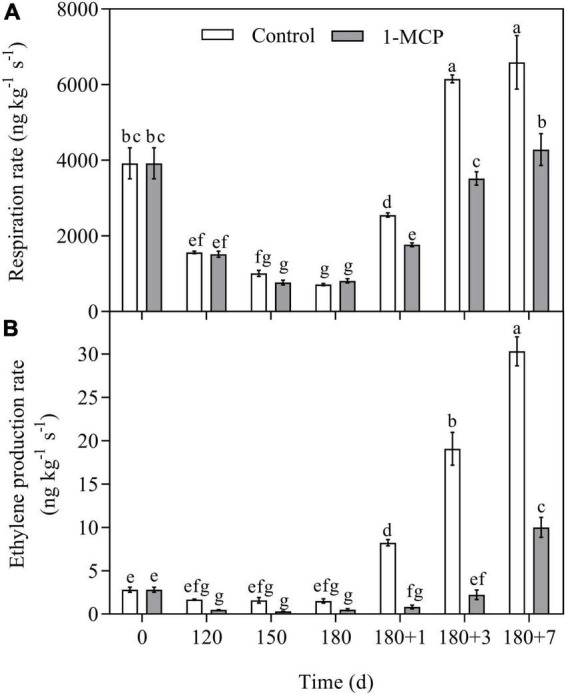
Effect of 1-MCP on respiration **(A)** and ethylene production **(B)** of “Yali” pear during cold storage and shelf life. 180 + 1, 180 + 3, 180 + 7 indicated Days 1, 3, 7 at shelf life after 180 days of cold storage, respectively. All data are expressed as means ± standard errors of triplicate samples. Different letters indicate significant differences (*P* < 0.05) by Duncan’s multiple comparison test.

The respiration and ethylene production rates during cold storage were lower than their initial values, but increased markedly when transferred to shelf life. 1-MCP had no significant impact on the respiration rate during cold storage, however, it dramatically reduced the respiration rate during shelf life (Figure 2A). In comparison with control, 1-MCP apparently reduced the ethylene production rate during cold storage and shelf life (Figure 2B).

### Expression of ethylene biosynthesis, receptor and response factor genes

**FIGURE 3:**
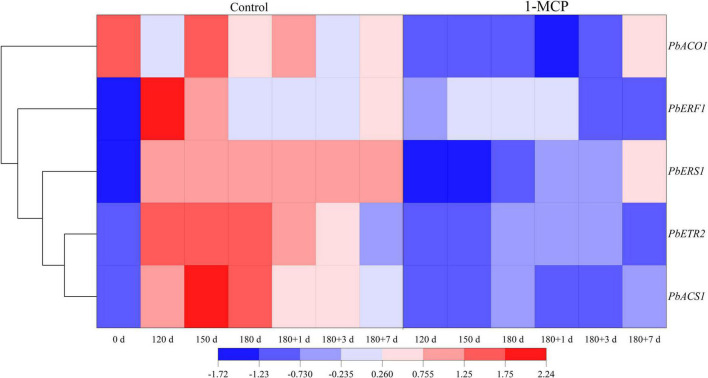
Effect of 1-MCP on the expression of ethylene synthesis genes (*PbACS1*, *PbACO1*), receptor genes (*PbETR2*, *PbERS1*), and ethylene response factor genes (*PbERF1*) of the peels in “Yali” pear during cold storage and shelf life. 180 + 1, 180 + 3, 180 + 7 indicated Days 1, 3, 7 at shelf life after 180 days of cold storage, respectively.

During cold storage, the expression of ethylene synthesis genes (*PbACS1*, *PbACO1*), ethylene receptors (*PbETR2*, *PbERS1*) increased significantly, while ethylene response factor (*PbERF1*) increased initially and then decreased in control. During shelf life, the expression of *PbACS1*, *PbACO1*, *PbETR2*, and *PbERS1* decreased to some extent, but that of *PbERF1* increased slightly. Cluster analysis showed that the gene expression patterns of *PbACS1*, ethylene receptors *PbETR2* and *PbERS1* were similar and that the downstream signals *PbERF1* were the same (Figure 3).

### Contents of α-farnesene, conjugated trienols and the related gene expression

**FIGURE 4:**
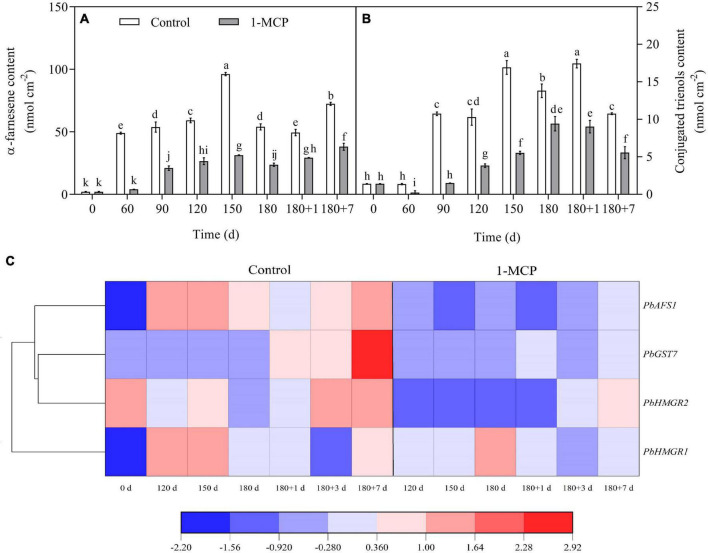
Effect of 1-MCP on α-farnesene **(A)** and conjugated trienols **(B)** contents and related gene expression **(C)** of the peels in “Yali” pear. 180 + 1, 180 + 7 indicated Days 1, 7 at shelf life after 180 days of cold storage, respectively. All data are expressed as means ± standard errors of triplicate samples. Different letters indicate significant differences (*P* < 0.05) by Duncan’s multiple comparison test.

The contents of α-farnesene and conjugated trienols in the fruit peel of control group reached a peak after 150 days of cold storage and then decreased. After 7 days of shelf life, the content of α-farnesene increased, but conjugated trienols decreased significantly. The trends of α-farnesene and conjugated trienols were similar between the 1-MCP treatment and the control group, while the contents of α-farnesene and conjugated trienols were markedly reduced for 1-MCP treatment group (Figures 4A,B).

During cold storage, the expression of *PbHMGR1* increased initially, then decreased at Day 180, and lastly increased at the end of shelf life. The expression amount of *PbHMGR1* was significantly upregulated by 1-MCP at Day 180 but decreased at other stages. The expression level of *PbHMGR2* showed clear variation during cold storage and decreased by 1-MCP. The expression of *PbGST7* changed slightly during cold storage, and was markedly upregulated during shelf life, and was significantly decreased by 1-MCP. The expression of *PbAFS1* decreased in the later stage of cold storage but then increased during shelf life, moreover, it was significantly inhibited by 1-MCP (Figure 4C).

### Contents of phenolic acids and flavonoids and the related synthesis genes expression

**FIGURE 5:**
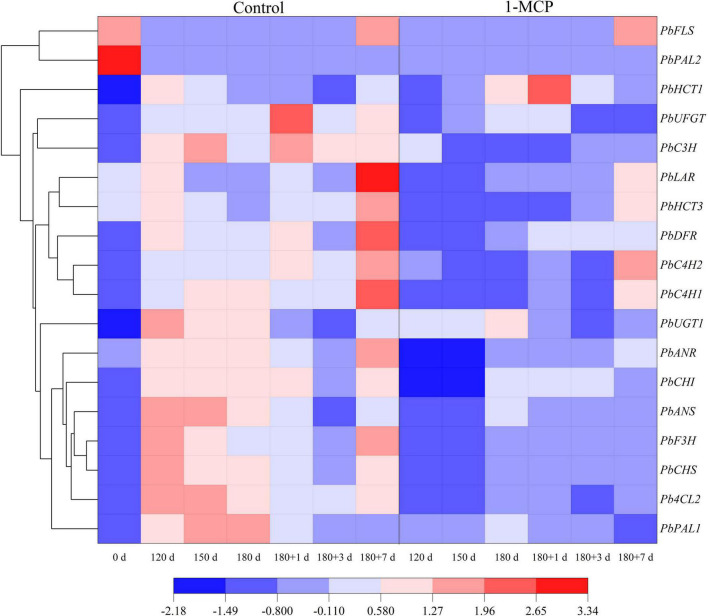
Effect of 1-MCP on phenolic acid and flavonoid synthesis-related genes expression of the peels in “Yali” pear. 180 + 1, 180 + 3, and 180 + 7 indicated Days 1, 3, and 7 at shelf life after 180 days of cold storage, respectively.

The results showed that phenolic acids of the peel mainly included chlorogenic acid, arbutin, and neochlorogenic acid. Flavonoids mainly included epicatechin, quercetin and proanthocyanidin B2. During cold storage stage, the phenolic acid content in the peels increased gradually, reached to the peak on the first day of shelf life, and then decreased. Treatment with 1-MCP significantly reduced the content of chlorogenic acid at Day 150, 180, and 180 + 1. Similarly, the content of epicatechin was significantly reduced by 1-MCP at Day 120, 150, 180, and 180 + 1. Unlike the above changes, the content of quercetin maintained at a higher level for the group treated with 1-MCP. The changes of other phenolic acids (e.g., arbutin) and flavonoids (e.g., kaempferol-3-o-glucoside) had fewer or no significant differences between the two groups (Table 1). During cold storage stage, except for *PbPAL2*, the expression levels of other genes (*PbPAL1*, *PbC4H1*, *PbC4H2*, *PbHCT3*, *Pb4CL2*, *PbC3H*, *PbHCT1*) were enhanced to different degrees, in which *PbPAL1* showed the greatest variation. At shelf life, the expression of *PbC4H1*, *PbC4H2*, and *PbHCT3* increased significantly, but was downregulated by 1-MCP. In contrast, the expression of the *PbHCT1* gene was inhibited by 1-MCP during cold storage but promoted at Day 180 and 180 + 1 (Figure 5).

**TABLE 1:** Effect of 1-MCP on contents of phenolic acids and flavonoids of the peels in “Yali” pear (mg kg^–1^).

	Day	Arbutin	Quininic acid	Chlorogenic acid	Epicatechin	Procyanidine B2	Procyanidine C1	Neochlorogenic acid	Quercetin	Rutin	Vanillic acid	Kaempferol-3-O-glucoside	Catechin
	0	1904.3 ± 20.42h	473.0 ± 1.76i	475.2 ± 8.76h	241.5 ± 4.16j	19.4 ± 1.64f	13.3 ± 0.78g	5.3 ± 0.19g	8.8 ± 0.15c	4.9 ± 0.35f	1.7 ± 0.15cd	1.1 ± 0.09g	1.0 ± 0.04h
Control	120	1927.4 ± 22.79h	637.7 ± 17.48fg	633.7 ± 19.54f	323.6 ± 12.78de	26.8 ± 1.55e	17.1 ± 2.38f	7.3 ± 0.08e	6.0 ± 0.13f	4.7 ± 0.30fg	1.8 ± 0.09cd	1.5 ± 0.09f	1.7 ± 0.04e
	150	2027.7 ± 63.89fg	649.4 ± 10.85ef	663.8 ± 6.96e	309.7 ± 11.74ef	27.8 ± 2.19e	19.7 ± 1.75ef	6.8 ± 0.30f	5.6 ± 0.13g	5.5 ± 0.27e	1.7 ± 0.18cd	1.7 ± 0.05de	1.7 ± 0.05e
	180	2114.5 ± 51.82def	707.2 ± 14.63d	688.9 ± 1.71cd	352.0 ± 15.89b	28.1 ± 0.93de	19.4 ± 2.59ef	8.0 ± 0.22d	6.2 ± 0.12f	7.2 ± 0.19a	1.8 ± 0.05cd	2.1 ± 0.04bc	2.0 ± 0.01b
	180 + 1	2556.9 ± 134.14a	787.4 ± 14.08a	775.6 ± 4.27a	398.2 ± 8.83a	39.5 ± 1.94a	26.4 ± 1.85ab	10.1 ± 0.34a	6.2 ± 0.09f	6.1 ± 0.39c	2.2 ± 0.05a	2.1 ± 0.06b	2.4 ± 0.02a
	180 + 3	2076.1 ± 42.87ef	723.2 ± 14.59cd	704.0 ± 8.59bc	334.3 ± 6.37cd	36.1 ± 2.76ab	23.4 ± 2.52 bcde	8.6 ± 0.08c	5.6 ± 0.07g	5.7 ± 0.31de	1.9 ± 0.05bc	1.9 ± 0.07c	1.8 ± 0.08d
	180 + 7	2216.5 ± 17.66c	701.0 ± 12.77d	675.1 ± 10.95de	285.9 ± 5.73g	39.7 ± 3.27a	27.0 ± 1.98ab	10.0 ± 0.25a	5.4 ± 0.08g	6.7 ± 0.07b	1.6 ± 0.15d	1.9 ± 0.03c	1.9 ± 0.07c
1-MCP	120	2148.0 ± 46.47cde	671.8 ± 16.88e	663.1 ± 18.86e	306.5 ± 10.91fg	37.5 ± 2.01a	25.1 ± 4.24abc	8.6 ± 0.08c	10.2 ± 0.20a	6.0 ± 0.14cd	2.0 ± 0.02ab	1.7 ± 0.09d	1.3 ± 0.09f
	150	1942.8 ± 5.48gh	620.2 ± 14.23g	610.7 ± 14.42g	292.9 ± 6.44gh	31.9 ± 1.99cd	21.7 ± 0.69cde	8.0 ± 0.16d	9.6 ± 0.20b	4.7 ± 0.21fg	1.7 ± 0.19cd	1.6 ± 0.19ef	1.1 ± 0.03g
	180	1865.5 ± 13.08h	591.7 ± 14.23h	589.2 ± 17.12g	258.4 ± 3.47i	28.4 ± 0.72de	16.9 ± 1.79f	6.8 ± 0.08f	8.4 ± 0.12d	4.0 ± 0.21h	1.6 ± 0.13d	1.5 ± 0.07ef	1.0 ± 0.06h
	180 + 1	2390.7 ± 36.32b	738.8 ± 15.81bc	722.4 ± 7.09b	348.9 ± 6.93bc	38.0 ± 1.31a	24.7 ± 1.67abcd	10.1 ± 0.22a	9.8 ± 0.16b	6.2 ± 0.16c	2.1 ± 0.07ab	2.1 ± 0.09bc	1.2 ± 0.02f
	180 + 3	2213.7 ± 33.82c	752.3 ± 6.55b	761.7 ± 12.85a	339.9 ± 6.37bc	39.6 ± 2.17a	27.7 ± 1.50a	9.0 ± 0.17b	8.7 ± 0.30c	4.5 ± 0.11g	2.1 ± 0.17ab	1.9 ± 0.02c	1.3 ± 0.02f
	180 + 7	2199.8 ± 59.22 cd	668.1 ± 24.99e	670.9 ± 24.84de	263.1 ± 10.71i	33.2 ± 3.88bc	20.8 ± 1.95def	8.4 ± 0.19c	7.8 ± 0.16e	5.0 ± 0.14f	1.7 ± 0.11d	2.6 ± 0.06a	1.3 ± 0.07f

180 + 1, 180 + 3, 180 + 7 indicated Days 1, 3, 7 at shelf life after 180 days of cold storage, respectively. All data are expressed as means ± standard errors of triplicate samples. Different letters indicate significant differences (*P* < 0.05) by Duncan’s multiple comparison test.

The expression levels of *PbCHS*, *PbUGT1*, *PbCHI*, *PbF3H*, *PbANS*, and *PbANR* were initially increased and then decreased during cold storage and at the end of shelf life, while those of *PbLAR*, *PbFLS*, *PbDFR*, and *PbUFGT* were increased to different degrees during cold storage and then decreased, but showed a huge increase at the end of shelf life (Day 180 + 7), especially *PbLAR*. And obviously, 1-MCP displayed effective inhibition to the expression of those genes (Figure 5). Nevertheless, the expression level of *PbFLS* was significantly enhanced by 1-MCP, while other genes were inhibited (Figure 5).

### Expression of *PbPPOs* and *PbLACs*

**FIGURE 6:**
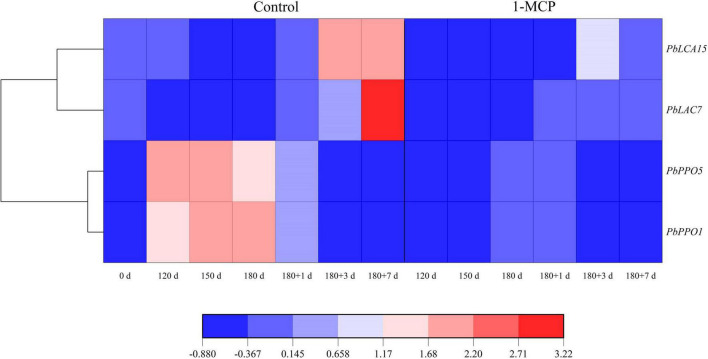
Effect of 1-MCP on the expression profiles of *PbPPOs* and *PbLACs* in the peels of “Yali” pear. 180 + 1, 180 + 3, and 180 + 7 indicated Days 1, 3, and 7 at shelf life after 180 days of cold storage, respectively.

During cold storage, the expression *of PbPPO1* and *PbPPO5* was significantly enhanced initially and then decreased until the end of shelf life. Differently, the expression levels of *PbLAC7* and *PbLAC15* did not show obvious changes, and even decreased to some extent during cold storage, but they were enhanced to different degrees during shelf life in the control group. However, it was observed that the expression levels of *PbPPO1*, *PbPPO5*, *PbLAC7*, and *PbLAC15* of 1-MCP-treated fruit were significantly lower than those in the control group at the same stage (Figure 6).

### Correlation and principal component analysis

The correlation analysis (Supplementary Figure 1) reveals that the superficial scald index had a significantly positive correlation with the contents of arbutin (*r* = 0.536*), quininic acid (*r* = 0.621*), chlorogenic acid (*r* = 0.650*), procyanidine B2 (*r* = 0.614*), neochlorogenic acid (*r* = 0.582*), kaempferol-3- o-glucoside (*r* = 0.708^**^), catechin (*r* = 0.552*), and conjugated trienols (*r* = 0.589*), and also with the expression levels of *PbERS1* (*r* = 0.627*), *PbGST7* (*r* = 0.820^**^), *PbC4H2* (*r* = 0.717^**^), and *PbUFGT* (*r* = 0.650), but had a significantly negative correlation with *PbPAL2* (*r* = −0.625*).

**FIGURE 7:**
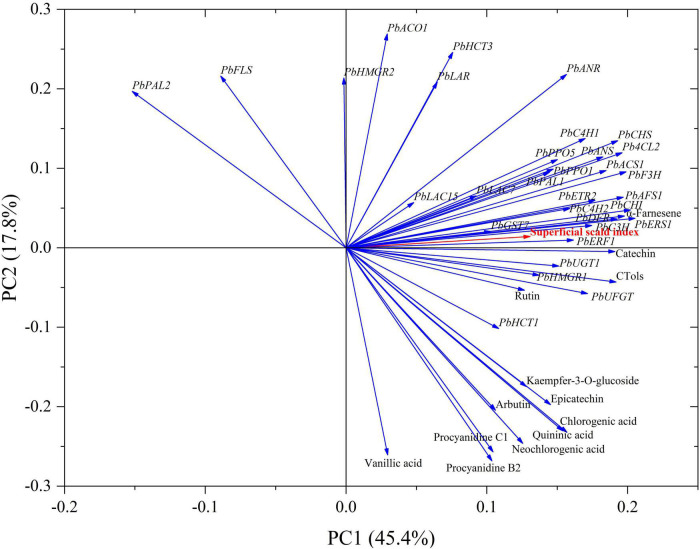
Loading plot of principal component analysis (PCA) of scald index, phenolics contents, and the related genes expression levels in the peels of the control group and 1-MCP-treated “Yali” pears. Arrows indicated the different variables.

Principal component analysis indicated that the total variability was explained by the first two principal components (PCs) with PC1 and PC2 accounting for 45.4 and 17.8% of the variation, respectively. It was shown that the contents of α-farnesene, CTols and catechin, and the expression levels of *PbERF1*, *PbC3H*, *PbERS1*, *PbDFR*, and *PbCHI* were clustered in the same interval with superficial scald index. The content of rutin, and the expression levels of *PbUGT1*, *PbUFGT*, *PbHMGR*, *PbAFS1*, *PbETR2*, and *PbC4H2*, also included the expression levels of *PbLAC7*, *PbPAL1*, *PbPPO1*, *PbPPO5*, *PbF3H*, *PbACS1*, *PbANS*, *Pb4CL2*, *PbCHS*, and *PbC4H1* were scatted in a cluster, this further confirmed the contribution of above-mentioned genes to the development of superficial scald (Figure 7).

## Discussion

1-Methylcyclopropene did not clearly affect the firmness and SSC of “Yali” pear (Figures 1A,B), which may be closely related to the slower softening characteristic of “Yali” pear (Wei et al., 2015). The ethylene production rate of the “Yali” pear was lower during cold storage and increased when they were transferred to shelf life. 1-MCP significantly reduced the respiration rate and ethylene production rate (Figure 2) and lowered the expression of the ethylene synthesis genes (*PbACS1*, *PbACO1*), receptor and signal transduction genes (such as *PbETR2*, *PbERS1*, and *PbERF1*) (Figure 3). The result is in line with previous studies (El-Sharkawy et al., 2003; Xie et al., 2016, 2017; Zhou et al., 2017), therefore, it was further demonstrated that 1-MCP could inhibit the expression of ethylene signal related genes in “Yali” pear. The correlation coefficient and loading plot (Figure 7 and Supplementary Figure 1) further confirmed that the superficial scald index had a significantly positive correlation with the expression level of *PbERS1*. Thus, the ethylene receptor *PbERS1* plays an essential role in the development of superficial scald.

The metabolism of α-farnesene is involved in the development of superficial scald, especially due to the accumulation of conjugated trienols (Lurie and Watkins, 2012; Ding et al., 2019). In the present study, we have showed that 1-MCP could significantly repress the accumulation of α-farnesene and conjugated trienols in the peels (Figures 4A,B). The scald showed serious symptoms after the reaching of peak level for α-farnesene and conjugated trienols (Figures 1C,D, 4A,B), which indicated that the accumulation of α-farnesene and conjugated trienols after long-term cold storage (180 days) was closely related to the onset of the scald. The *HMGR* and *AFS1* genes were strongly associated with the α-farnesene and development of scald (Zhou et al., 2017). Thus, the variation in α-farnesene was consistent with the expression of *PbHMGR* and *PbAFS1* in peels. Additionally, GST and GPX play a role in oxidative damage and cell senescence (Chen et al., 2004; Gill and Tuteja, 2010; Hui et al., 2016). The present study further revealed that the expression of *PbGST7* was enhanced with the development of scald in the “Yali” pear, which might affect the change of the content of conjugated trienol. In addition, the scald index was significantly correlated with the expression level of *PbGST7* (*r* = 0.820, Supplementary Figure 1). The results indicate that *PbHMGR*, *PbAFS*, and *PbGST* are closely related to the development of scald. According to the PCA results (Figure 7), the contents of α-farnesene, conjugated trienols and the expression levels of *PbERF1*, *PbERS1* were clustered together, suggesting that ethylene had a regulatory effect on the accumulation of α-farnesene and conjugated trienols.

Phenolics in cells are generally recognized to be oxidized to quinones and cause tissue browning. Phenolic oxidation catalyzed by PPO contributed to the development of superficial scald (Busatto et al., 2014; Niu et al., 2018; Lindo-García et al., 2020). It has been reported that the accumulation of chlorogenic acid and epicatechin was closely related to the development of the superficial scald (Busatto et al., 2014; Gong et al., 2018; Cebulj et al., 2020). This study further demonstrated that the development of the scald in “Yali” pear was accompanied by the accumulation of chlorogenic acid and epicatechin (Table 1). Additionally, the development of the scald was accompanied by the increase in expression levels of *PbPPO1, PbPPO5*, *PbLAC7*, and *PbLAC15*, in which were lowered by 1-MCP (Figure 6). In addition, the expression of *PbETR2* and *PbACS1* was clustered in a same interval with *PbLAC7*, *PbPPO1*, and *PbPPO5* (Figure 7), indicating that the expression of *PbETR2* and *PbACS1* was closely related to that of *PbLAC7*, *PbPPO1*, and *PbPPO5*, and therefore, the enzymatic reaction catalyzed by PPO and LAC was reduced and the occurrence of the scald was inhibited subsequently through ethylene inhibition by 1-MCP.

Previous studies have shown that 1-MCP could restrain the expression of *PAL* and *C3H*, and reduce the occurrence of superficial scald (Busatto et al., 2014, 2018; Du et al., 2017), which are in consistent with our results (Figures 1C, 4). C3H and ANR are the key enzymes that promote the synthesis of chlorogenic acid and epicatechin, respectively (Henry-Kirk et al., 2012). 1-MCP markedly reduced the transcription of *PbC3H* and *PbANR* (Figure 5), which might result in the lower level of chlorogenic acid and epicatechin in peels (Table 1). We also noticed that treatment by 1-MCP enhanced the content of quercetin (Table 1), since FLS is the key enzyme for quercetin synthesis (Henry-Kirk et al., 2012). Therefore, the upregulation of the expression of *PbFLS* by 1-MCP may be an important factor in accelerating the synthesis of quercetin. In contrast, the content of flavonoids decreased at the onset and development of scald in apples (Cebulj et al., 2020). This further indicated that quercetin might play a role at the beginning of scald, which should be fully studied later.

## Conclusion

In conclusion, superficial scald was found after 180 days of cold storage, and the symptoms became serious at shelf life, which was accompanied by higher respiration and ethylene production rates, in combination with the accumulation of conjugated trienols, chlorogenic acid and epicatechin in the peels of “Yali” pear after a long-term of cold storage. 1-MCP significantly decreased superficial scald index with the lower contents of α-farnesene, conjugated trienols, chlorogenic acid, epicatechin, catechin and rutin in the peels, and meanwhile reduced the expression levels of genes associated with ethylene biosynthesis (*ACS1*, *ACO1*), receptors and signal transduction (*ETR2*, *ERS1*, *ERF1*), α-farnesene metabolism (*AFS1*, *HMGR2*, *GST7*), and phenolic synthesis (*PAL1*, *C4H1*, *C4H2*, *HCT3*, *4CL2*, *C3H*) as well as *PPO1, PPO5*, and *LAC7*, except that increased the expression of *FLS* gene. In addition, *PPO1* and *PPO5* were associated with the onset of superficial scald, and the *LAC7* gene was closely related to the development of superficial scald in “Yali” pear.

## Data availability statement

The original contributions presented in this study are included in the article/Supplementary material, further inquiries can be directed to the corresponding author.

## Author contributions

JG, JH, and YC conceived the work. JH, YF, YC, and MW conducted the experiments. JH, YF, YC, MW, and JG contributed to analyses and interpretation. JH, MW, and JG wrote the manuscript. All authors contributed to the article and approved the submitted version.
