# Supplementary material for: A comprehensive insight on the main physiological biochemical and related genes expression changes during the development of superficial scald in “Yali” pear
Source: Front Plant Sci. 2022 Sep 2;13:987240. doi: 10.3389/fpls.2022.987240 (PMC9478120; doi:10.3389/fpls.2022.987240)
Supplement: Supplementary file 1 [file Table_1.docx]

**Supplementary TABLE 1|**The sequences and reference source of qRT-PCR primers

| Gene  Annotation | GenBank Accession ID | Primer sequence (5’ to 3’) | References |
| --- | --- | --- | --- |
| *ACS1*  1-aminocyclopropane-1-carboxylate synthase | XM_018643584 | GAGATTGTACCCATTCACTGCA  AGGAGGTAGAGTTCGTTTCTGGT | Li et al., 2018 |
| *ACO1*  1-aminocyclopropane-1-carboxylate oxidase | XM_009380726 | TCAGTGGCATCAATGGTAGTG  CATATTTTCCAGCACACTCAGTG | Li et al., 2018 |
| *PbETR2*  ethylene receptor 2 | XM_009350325 | GATCCTGGAGAATCATCAGAGC  GCAGTTACAATGCAACCAAGC | Zhou et al., 2017 |
| *PbERS1*  ethylene response sensor 1 | XM_009380469 | CGTTGCATTCATCGTCAAACTG  TCGATGAACTTGCGCCAAATCG | Zhou et al., 2017 |
| *PbERF1*  Ethylene response factor 1 | XM_009364438 | TCCCTACTCACAAAGCCCAAG  CGAGGCTAAAAGCATCACG | Li et al., 2018 |
| *PbHMGR1*  3-hydroxy-3-methylglutaryl-coenzyme A reductase | XM_009360626 | GTTCTCACTGCATTACCATG  TCAGACAAGCGGATTGAGATG | Zhou et al., 2017 |
| *PbHMGR2*  3-hydroxy-3-methylglutaryl-coenzyme A reductase | XM_009371497 | CAGTTGGAGGAGGAACCCAG  AGTTTGAACCGGGTGAGTC | Zhou et al., 2017 |
| *PbAFS1*  alpha-farnesene synthase | GQ421155 | AATGGTTGGAACCAAGTATTACC  GAAACTGATGATGAATCGCATCC | Zhou et al., 2017 |
| *PbGST7*  glutathione S-transferase | XM_009372621 | ATGGTTCCGGTGTTTGTCCA  GAGGGTCATTTGGCAGCAGA | Zhou et al., 2017 |
| *PbPAL1*  phenylalanine ammonia-lyase 1 | GU906268 | GACGCACAAGTTGAAGCACC  TTTGACATAAGAGCTGCCATCC | Zhou et al., 2020 |
| *PbPAL2*  phenylalanine ammonia-lyase 2 | GU906269 | ACCAAAAGAAGGGCTTGCCT  AAAGCGCTTGTGTGTTAGCG | Zhou et al., 2020 |
| *PbC4H1*  trans-cinnamate 4-monooxygenase | XM_009376113 | AACTTCGAGCTTCTGCCTCC  CCCCAAGCATCAATCTACGC | Zhou et al., 2020 |
| *PbC4H2*  trans-cinnamate 4-monooxygenase | XM_009356593 | CAAGCACACGGGCTACAAC  GATCGACCACAACGTGGTTT | Zhou et al., 2020 |
| *Pb4CL2*  4-coumarate--CoA ligase 2 | XM_009353551 | AATTCAAAGGCTTCCAAGTGC  CACTAGACCGAACCACAAATGC | He et al., 2017 |
| *PbHCT1*  hydroxycinnamoyl-coenzyme A shikimate/quinatehydroxycinnamoyl transferase 1 | JQ280303 | CCCCCTCCAGTCTGACCA  CCAATGAAAACACAAACACGTC | Zhou et al., 2020 |
| *PbHCT3*  shikimate O-hydroxycinnamoyltransferase | NM_001302311 | ACGATATGCGTGAGAAGTTTATCC  AGCACTAGGAAAGAAACACGACC | He et al., 2017 |
| *PbC3H*  cytochrome P450 98A2 | XM_009355500 | TTACAAGCCGTGCCTACTCC  CACAAAAACAAGAAACACTACGG | Zhou et al., 2020 |
| *PbCHS*  chalcone synthase | NM_001319814 | TGTGCGAGTACATGGCTCC  GGTGCAAAAGACCAAGTGG | Fischer et al., 2007 |
| *PbUGT1*  UDP-glucose glucosyltransferase | XM_009371845 | GGTCGCTCGTTTAATCTGTCG  GAGATCCCATATTTTGCTGTCT | Jugdé et al., 2008 |
| *PbCHI*  chalcone--flavonone isomerase | XM_009350800 | CCATTTTCCTGCAACTTCTGC  CCATTTCTTGATGTTCTCCACC | Qian et al., 2014 |
| *PbF3H*  naringenin,2-oxoglutarate 3-dioxygenase | XM_009365221 | CAGAAGCCATGGGGTTGGAT  CCAAGAGTTAGGTCGGGCTG | Qian et al., 2014 |
| *PbFLS*  flavonol synthase/flavanone 3-hydroxylase | XM_009343391 | GCCGGTGGTGATAACTTGGA  TGAACATCGTTGGGGACGAG | Fischer et al., 2007 |
| *PbDFR*  bifunctional dihydroflavonol 4-reductase/flavanone 4-reductase | XM_018642874 | ATGGACTTTGAATCCAAGGACC  CTCCACATTCACAGTTCCTGC | Fischer et al., 2007 |
| *PbLAR*  leucoanthocyanidin reductase | XM_009357917 | TCCAACAGTTTGGGGGTGAG  TCCAGTTTCCTTGTCCAGCG | Fischer et al., 2007 |
| *PbANS*  leucoanthocyanidin dioxygenase | XM_009364555 | TGCGTTCCAAATTCCATCG  CTGCCCATGAAATCCTCACC | Fischer et al., 2007 |
| *PbANR*  anthocyanidin reductase | XM_009370102 | ACAACCCATCTCAAAGAAGACG  GTAGCCCTTCTGGAGCAGC | Fischer et al., 2007 |
| *PbUFGT*  UDP-glucose flavonoid 3-O-glucosyltransferase 7 | XM_009356440 | GGTACATTTCTGGATGTGATGACG  GGCTCTGCCTCTCATTCCCT | Qian et al., 2014 |
| *PbPPO1*  polyphenol oxidase I | HQ729709 | TCCCTACTCACAAAGCCCAAG  GACCTCCAAGACCAAGAAGCA | Zhou et al., 2020 |
| *PbPPO5*  polyphenol oxidase V | GU906266 | ACCAAAACAAAAACCATTCCAC  CAGCCACTCCACCATACAGG | Zhou et al., 2020 |
| *PbLAC7*  laccase-7 | XM_009348720 | TCATCAACCCCCATTATGC  GAACATGTGCTCGTCCACC | Gong et al., 2018 |
| *PbLAC15*  laccase-15 | XM_009353565 | ACTGCCATACTCCAATACCACG  TTATCAGCCAAGCTTTTGAGTTTAC | Gong et al., 2018; Zhai et al., 2021 |
| *PbACTIN2* | GU830959 | GGACATTCAACCCCTCGTCT  ATCCTTCTGACCCATACCAACC | Cheng et al., 2015 |

(Cheng et al., 2015; Fischer et al., 2007; Gong et al., 2018; He et al., 2017; Jugdé et al., 2008; Li et al., 2018; Qian et al., 2014; Zhai et al., 2021; Zhou et al., 2020)
